# MEMS Accelerometer Noises Analysis Based on Triple Estimation Fractional Order Algorithm

**DOI:** 10.3390/s22020527

**Published:** 2022-01-11

**Authors:** Michal Macias, Dominik Sierociuk, Wiktor Malesza

**Affiliations:** Institute of Control and Industrial Electronics, Warsaw University of Technology, ul. Koszykowa 75, 00-662 Warsaw, Poland; dominik.sierociuk@pw.edu.pl (D.S.); wiktor.malesza@pw.edu.pl (W.M.)

**Keywords:** fractional calculus, fractional Kalman filter, estimation of fractional order systems, fractional order noise

## Abstract

This paper is devoted to identifying parameters of fractional order noises with application to noises obtained from MEMS accelerometer. The analysis and parameters estimation will be based on the Triple Estimation algorithm, which can simultaneously estimate state, fractional order, and parameter estimates. The capability of the Triple Estimation algorithm to fractional noises estimation will be confirmed by the sets of numerical analyses for fractional constant and variable order systems with Gaussian noise input signal. For experimental data analysis, the MEMS sensor SparkFun MPU9250 Inertial Measurement Unit (IMU) was used with data obtained from the accelerometer in *x*, *y* and *z*-axes. The experimental results clearly show the existence of fractional noise in this MEMS’ noise, which can be essential information in the design of filtering algorithms, for example, in inertial navigation.

## 1. Introduction

Micromachined Electrical Mechanical Systems (MEMS) are mechanical and electro-mechanical devices made using microfabrication techniques. MEMS technology allows to build miniature (e.g., inside integrated circuits) sensor and actuators which have been employed in many different areas such as medicine [1], biology [2], chemistry [3], aerospace [4], and motor vehicles [5]. A crucial area in which MEMS sensors are used is inertial navigation systems (INS) [6,7] based on double integration of body acceleration processes based on accelerometers and gyroscopes measurements. Due to the double integration action, high accuracy and precision of acceleration measurement are essential because noises (especially biases) are double-integrated and rapidly increase navigation errors. That is why modelling noises, biases, and general dynamics of MEMS sensors are essential. An article [8] uses, for example, an advanced type of recurrent neural network to model some parts of non-modeled MEMS gyroscope dynamics and apply this network into fractional order sliding mode control. In [9], analysis of noises in MEMS sensors is presented. The article [10] presents a mathematical modelling MEMS sensors dynamics, including modelling the noises. Presented in the mentioned work, the model is very complex and underlines the impact of temperature noises. The impact of thermal noise is also presented in [11]. What is important, as it will be discussed later, is that diffusive systems, in particular heat transfer processes, can be very efficiently modelled using fractional order calculus. Moreover, in [12], a fractional order algorithm was effectively used to estimate the bias of the MEMS sensor. That is why in this paper, we will use a fractional order estimation algorithm to identify noises of MEMS’ sensor as a fractional order noise.

The fractional calculus (FC) itself, is an extension of traditional differential and integral calculus. The differential orders in FC can be real or even complex numbers. The fractional derivative appeared for the first time in the correspondence between Leibniz and l’Hôpital in 1695, and thereby, it appeared almost simultaneously with the integer order calculus. The theoretical background for this calculus can be found in already classic works such as [13,14,15,16,17,18], as well as (with some applications) in relatively recently published books [19,20,21].

In contradiction to integer order derivatives, the fractional order derivatives depend not only on local time conditions but also on the whole past of the function [22]. This specific property has an advantage when the analysed dynamics possess a long-term memory nature, and thus, past values of the function are taken into account. The FC was found to be especially efficient in modelling diffusive systems [23,24,25,26]. For example, in the heat transfer process of the solid beam, it is possible to describe dynamics between temperature and heat flux at the desired point as a half order integral. When the heated material is not solid (heterogeneous), the order of the integration can be different by half, as it was presented in [23].

The FC was also recognised as an efficient tool in signal processing to design new types of filters and new tools for signal analysis. Some applications of fractional order calculus to signal processing were presented in [17,27,28,29].

It may happen that using constant order operators applied for some complex dynamic processes may be unsatisfactory, for example, diffusion processes in the porous (inhomogeneous or heterogeneous) environment, where the structure may vary in time [24]. In such a case, the fractional variable order (FVO) operators have to be used. To give a deeper insight into FVO calculus, four switching schemes, equivalent to four definitions of FVO derivatives, are presented in [30,31,32]. The switching strategies introduced, given unambiguously, classify and identify ways of changing the order of derivatives (integrals). Based on those switching schemes, it is possible to categorise fractional order derivatives according to their behaviour and intrinsic properties.

For FVO systems, it was also a generalised Kalman Filter obtaining Variable Order Kalman Filter [33] applied for estimation of fractional variable order state-space systems over a lossy network. A generalisation of the Improved Fractional Kalman Filter (ExFKF) for variable order discrete state-space systems is presented in [34], where the results are used for estimation and smoothing actions.

When the uncorrelated noise (like white noise) passes through a dynamical system, the dynamically correlated noise (coloured noise) is obtained. When the dynamics contain fractional order, the fractional noise is obtained. Article [35] presents an introduction to fractional order noises (the noises obtained by applying uncorrelated white noise to fractional order dynamics).

In [36], fractional signal processing methods were used to electrochemical noise of corrosion processes in stainless steel. It has been shown there that conventional (integer order) methods failed to sufficiently distinguish between electrochemical noise signals obtained from different solutions, and the use of fractional Fourier transforms turned out to be a powerful tool that can better describe the corrosion processes of the electrode.

Article [37] presents estimation schemes for discrete fractional and integer order state-space systems with fractional order coloured noise. Better estimates of the state vector were obtained there thanks to the additional information about noise dynamics used by the proposed estimation algorithm.

Article [38] presents an adaptive filtering approach to filter the noise from MEMS measurements, where an adaptive Kalman filter was derived from the integer order dynamic noise model.

In [39], modelling results of temperature sensor noise were presented. The identification algorithm is based on numerical minimisation of dynamical correlation of identified source noise with the Least Square algorithm. The results presented show that the noise order of the temperature sensor is fractional. The used algorithm assumed a situation in which the only evident fractional order noise is analysed. However, in the real plant, we would rather have a situation when the measured noise is a combination of dynamically correlated and uncorrelated noises.

Interesting results showing the source of noises in MEMS, both of a mechanical and electrical nature, and in particular the impact of thermal-noise on bifurcation MEMS sensors, were presented in [11]. The authors proposed a stochastic integer order model of the sensor that takes these noise sources into account.

In [40], the Triple Estimation algorithm (TEA) for state vector, order, and system’s parameters estimation was proposed and described in detail. In this paper, we will present the TEA’s application to estimate noise parameters for a case when noise is a combination of correlated and uncorrelated dynamically noises. Moreover, we will also present the analysis results of a real plant noise obtained from MEMS sensor.

In [12], Fractional Kalman algorithm in two versions has been used to improve measurement results from MEMS sensors. The results of MEMS noises modelling obtained in our article could explain why Fractional Order Kalman Filter was more efficient in this case.

Thus, the main novelty of this paper is identifying and analysing the accelerometer noises built-in MEMS technology. We have conducted sets of experiments based on real plant data to show the fractional dynamic of the investigated sensor. The paper also describes the identification process of fractional order noises and problems connected with this action. The obtained results present the ability of the TEA to model the noises from real plants. All of these issues make a new contribution to this research topic.

The paper is organised as follows: Section 2 recalls elements of FC and fractional noises. In Section 3, the TEA is presented. Section 4 describes a method for application TEA to fractional noise identification. Finally, Section 5 presents results of using the TEA to model noises obtained from MEMS accelerometer.

## 2. Fractional Calculus and Fractional Noises

In FC, the three most popular definitions of fractional constant order integral and derivative are used, namely, Grünwald–Letnikov, Riemann–Liouville, and Caputo. These definitions possess different properties and may be applied in various areas of engineering.

In this paper, we use the Grünwald–Letnikov definition, which is usually used in discrete systems, as a base for FVO difference definition. Due to the application nature of this work, we will use a discrete approximation of the Grünwald–Letnikov derivative with a finite (not going to zero) sampling time *h*. Hence, we have constant order difference definition

(1) Δkα0xk≡∑j=0k1hα(−1)jαjxk−j,

where

αj≡1forj=0,α(α−1)…(α−j+1)j!forj>0,

α∈R is a fractional order and *h* is a time sampling.

In our paper, we will use the following FVO type of difference:

(2) Δkαk0Axk≡∑j=0k(−1)jhαkαkjxk−j,

where $\alpha_{k}\in R$ is FVO.

### Fractional Noise

The time-correlated (coloured) noises are the noises that contain a dynamical correlation between the noise’s samples. Such noises can be obtained when some noise (uncorrelated) is passed through dynamical systems. For example, the electromagnetic field noise can induct some current in an electronic circuit, leading to some dynamically correlated noise in voltage because of some dynamic between current and voltage. When the order of the dynamics is an integer, we will have a dynamically correlated integer order noise, which the following relation can describe:

(3) $$x_{k+1}=fx_{k}+\omega_{k},$$

where $x_{k}$ is a time-correlated noise, and $\omega_{k}$ is an uncorrelated noise for example white Gaussian noise.

When the dynamics of the system are fractional, for example, in temperature transport (for ideal beam temperature is half order integral of heat flux [23]), the uncorrelated heat flux noise can lead to fractional order dynamically correlated noise in temperature. The coloured fractional order noise is given as follows

(4) Δk+1α0xk+1=fxk+ωk

(5) xk+1=hαΔk+1α0xk+1−∑j=1k+1(−1)jαjxk−j+1,

where $x_{k}$ is a fractional coloured noise, α is an order of the noise, and $\omega_{k}$ is an uncorrelated noise.

For the case when the fractional order of the dynamical system changes in time (for example, when the structure of heated medium changes in time [24]), the FVO noise will appear. Depending on the order switching manner, different definitions can describe such dynamics. For example, for A-type definition, we will have the following FVO noise dynamics:

(6) Δkαk+10Axk+1=fxk+ωk

(7) xk+1=hαk+1Δkαk+10Axk+1−∑j=1k+1(−1)jαk+1jxk−j+1.

Identification of the fractional noise in a real application is a complex process because we do not know the order and system parameters of the noise. We also do not have information about dynamically uncorrelated source noise. In [39], identification algorithm for fractional noise was presented, however, under the assumption that output noise is the only evident fractional order noise. In experimentally obtained noises, we would instead acquire a combination of dynamically correlated and uncorrelated noises

(8) $$y_{k}=x_{k}+\nu_{k}.$$

That is why in this article, we use a Triple Estimation algorithm to identify parameters of fractional order noises.

## 3. Triple Estimation Algorithm

The TEA allows estimating state vector, system parameters, and fractional order simultaneously. The main idea of this algorithm is to separate states, parameters, and orders estimation processes. This separation allows a better adjustment of used filters, making it possible to obtain better estimation results. Detailed introduction of TEA was presented in [40].

The TEA will be defined for the following linear Discrete Fractional Variable Order State-Space (DFVOSS) A-type system [41]:

(9) Δk+1αk+10Axk+1=Axk+Buk+ωk,

(10) xk+1=hαk+1Δk+1αk+10Axk+1−∑j=1k+1(−1)jαk+1jxk−j+1,

(11) $$\begin{array}{ccc} y_{k} & = & Cx_{k}+\nu_{k}\;, \end{array}$$

where $u_{k}\in R^{d}$ is a system input; $y_{k}\in R^{p}$ is a system output; $A\in R^{N\times N}$, $B\in R^{N\times d}$, and $C\in R^{p\times N}$ are the state system, input, and output matrices, respectively; $x_{k}\in R^{N}$ is a state vector; *N* is a number of state equations.

In general, the TEA can be treated as a method for simultaneous states, parameters, and order estimation for fractional order systems. Moreover, separation of order and system parameters estimation processes allows better algorithm parameters tuning because we can separately tune parameters for order and system parameters filters.

**Figure 1:**
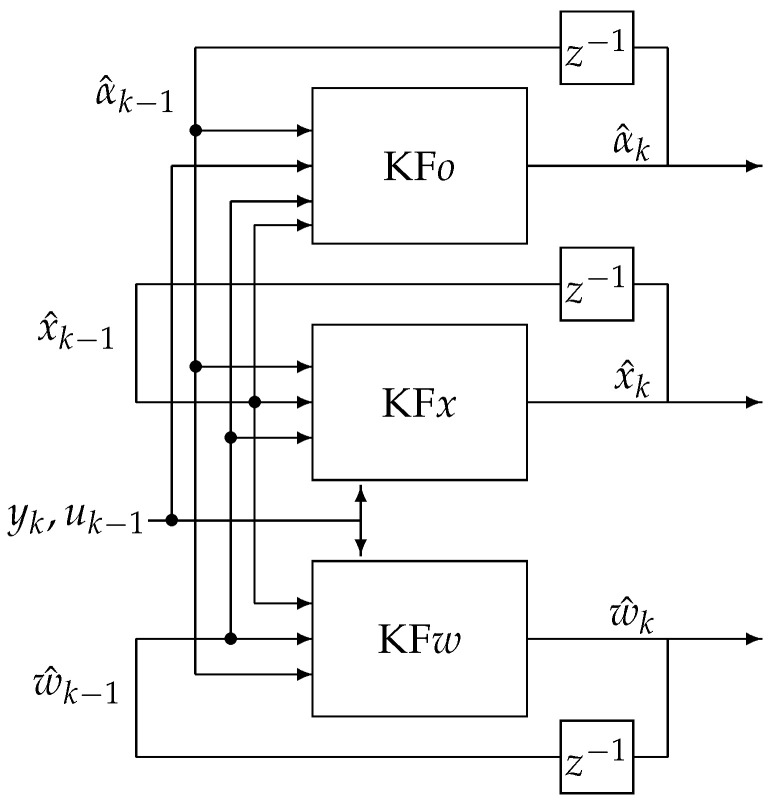
The Triple Estimation Algorithm scheme.

In the TEA process, the FVO, state variables and parameters estimation is divided into three estimation actions (filters). The first filter, KF*x*, estimates the state variables vector ${\hat{x}}_{k}$ based on order and system parameters estimates from other filters KF*o* and KF*w*, respectively. The second one, KF*w*, estimates the vector of system parameters ${\hat{w}}_{k}$ based on state variable and order estimates obtained in the remaining two filters KF*x* and KF*o*, respectively. The third filter, KF*o*, estimates the FVO with the knowledge of state variable and system parameters from filters KF*x* and KF*w*, respectively. The scheme of the TEA is given in Figure 1.

### 3.1. Order Estimation Filter KFo

Because the order estimation problem is highly non-linear (due to (αk,ij)) relations in obtaining state update process), as the KF*o* filter, the Unscented Fractional Variable Order Kalman Filter is used. The order changing dynamics is assumed to be a constant

(12) $$\alpha_{k+1}=\alpha_{k}+\omega_{k}^{o},$$

where $\omega_{k}^{o}$ is a noise with variance given by matrix $Q_{k}^{o}$. The matrix $Q_{k}^{o}$ represents our knowledge of how big fluctuations in time we are assumed. The bigger the value of this matrix, the more the algorithm will spread estimation error to modify the order.

The KF*o* algorithm equations are given as follows:

(13) $$\begin{array}{ccc} {\tilde{\alpha }}_{k} & = & {\hat{\alpha }}_{k-1}, \end{array}$$

(14) $$\begin{array}{ccc} {\tilde{P}}_{k}^{o} & = & {\hat{P}}_{k-1}^{o}+Q_{k-1}^{o}, \end{array}$$

(15) $$\begin{array}{ccc} {\tilde{\alpha }}_{k} & = & \left[\begin{array}{cc} {\tilde{\alpha }}_{k} & {\tilde{\alpha }}_{k}\pm {\left(\sqrt{\left(L+\lambda \right){\tilde{P}}_{k}^{o}}\right)}_{i} \end{array}\right], \end{array}$$

(16) $$\begin{array}{ccc} \Delta^{{\tilde{\alpha }}_{k,i}}{\tilde{\chi }}_{k,i}^{o} & = & A\left({\hat{w}}_{k-1}\right){\hat{x}}_{k-1}+Bu_{k-1}, \end{array}$$

(17) χ˜k,io=hα˜k,iΔα˜k,iχ˜k,io−∑j=1k(−1)jα˜k,ijx^k−j,

(18) $$\begin{array}{ccc} {\tilde{Y}}_{k,i}^{o} & = & C{\tilde{\chi }}_{k,i}^{o}, \end{array}$$

(19) $$\begin{array}{ccc} {\tilde{y}}_{k}^{o} & = & \sum_{i=0}^{2L}W^{\left(m\right)}{\tilde{Y}}_{k,i}, \end{array}$$

(20) $$\begin{array}{ccc} P_{y_{k}y_{k}}^{o} & = & \sum_{i=1}^{2L}W_{i}^{\left(c\right)}\left[{\tilde{Y}}_{i,k}-{\tilde{y}}_{k}\right]{\left[{\tilde{Y}}_{i,k}-{\tilde{y}}_{k}\right]}^{T}+R^{o}, \end{array}$$

(21) $$\begin{array}{ccc} P_{\alpha_{k}y_{k}}^{o} & = & \sum_{i=1}^{2L}W_{i}^{\left(c\right)}\left[{\tilde{\alpha }}_{i,k}-{\tilde{\alpha }}_{k}\right]{\left[{\tilde{Y}}_{i,k}-{\tilde{y}}_{k}\right]}^{T}, \end{array}$$

(22) $$\begin{array}{ccc} K_{k}^{o} & = & P_{\alpha_{k}y_{k}}^{o}{\left(P_{y_{k}y_{k}}^{o}\right)}^{-1}, \end{array}$$

(23) $$\begin{array}{ccc} {\hat{\alpha }}_{k} & = & {\tilde{\alpha }}_{k}+K_{k}^{o}\left(y_{k}-{\tilde{y}}_{k}^{o}\right), \end{array}$$

(24) $$\begin{array}{ccc} P_{k}^{o} & = & {\hat{P}}_{k}^{o}-K_{k}^{o}P_{y_{k}y_{k}}^{o}K_{k}^{o}, \end{array}$$

(25) $$\begin{array}{ccc} Q_{k}^{o} & = & \left(1-\delta^{o}\right)Q_{k-1}^{o}+\delta^{o}\left(K_{k}^{o}\right)\left(y_{k}-{\tilde{y}}_{k}^{o}\right){\left(y_{k}-{\tilde{y}}_{k}^{o}\right)}^{T}{\left(K_{k}^{o}\right)}^{T}, \end{array}$$

where ${\left(\sqrt{\left(L+\lambda \right)P_{k}}\right)}_{i}$ is *i*-th column of matrix square root (e.g., Cholesky factorisation), *L* is a dimension of estimated state vector (2L+1 is a number of sigma points) and coefficients of Unscented transformation *W* are equal to

(26) $$\begin{array}{ccc} W_{0}^{\left(m\right)} & = & \lambda /(L+\lambda ), \end{array}$$

(27) $$\begin{array}{ccc} W_{0}^{\left(c\right)} & = & \lambda /\left(L+\lambda \right)+\left(1-A^{2}+B\right), \end{array}$$

(28) $$\begin{array}{ccc} W_{i}^{\left(m\right)} & = & W_{i}^{\left(c\right)}=1/\left(2\left(L+\lambda \right)\right), \end{array}$$

where $\lambda =A^{2}\left(L+\kappa \right)-L$, A is a coefficient describing width of point expansion during the transformation (in literature is obtained in the range 1≤A≤1e−4, usually denoted as α, but in this article, because of using order α this notation has been changed); κ is an additional scaling coefficient usually chosen as 3-L; B is a coefficient that corresponds with our knowledge about type of noise, for Gaussian noise is chosen as B=2 (in literature usually denoted as β). The δ coefficient is a “forgetting factor” according to Robbins–Monro stochastic approximation scheme for estimating the innovations (see [42] p. 240). The initial values of matrix $P_{0}^{o}$ represent our a’priori knowledge about error in choosing initial value of order $\alpha_{0}$ (we assume, the initial value is different from the original).

### 3.2. State Estimation Filter KFx

As the KF*x* Filter, the Fractional Variable Order Kalman Filter algorithm is used because of the linearity of the state estimation sub-process. This filter is given as follows:

(29) Δk+1α^k0Ax˜k+1=A(w^k−1)x^k+Buk,

(30) x˜k+1=hα^kΔk+1α^k0Ax˜k+1−∑j=1k+1(−1)jα^kjx^k+1−j,

(31) $$\begin{array}{ccc} {\tilde{P}}_{k} & = & \left(h^{{\hat{\alpha }}_{k}}A\left({\hat{w}}_{k-1}\right)+{\hat{\alpha }}_{k}\right)P_{k-1}{\left(h^{{\hat{\alpha }}_{k}}A\left({\hat{w}}_{k-1}\right)+{\hat{\alpha }}_{k}\right)}^{T} \end{array}$$

(32) +Qk−1+∑j=2kα^kjPk−jα^kjT,

(33) $$\begin{array}{ccc} K_{k} & = & {\tilde{P}}_{k}C^{T}{\left(C{\tilde{P}}_{k}C^{T}+R_{k}\right)}^{-1}, \end{array}$$

(34) $$\begin{array}{ccc} {\hat{x}}_{k} & = & {\tilde{x}}_{k}+K_{k}\left(y_{k}-C{\tilde{x}}_{k}\right), \end{array}$$

(35) $$\begin{array}{ccc} P_{k} & = & \left(I-K_{k}C\right){\tilde{P}}_{k}, \end{array}$$

where initial conditions are

(36) $$x_{0}\in R^{N},\;P_{0}=E\left[\left({\tilde{x}}_{0}-x_{0}\right){\left({\tilde{x}}_{0}-x_{0}\right)}^{T}\right],$$

and $\nu_{k}$ and $\omega_{k}$ are assumed to be independent with zero expected value.

### 3.3. Parameters Estimation Filter KFw

For KF*w* filter another Unscented Fractional Variable Order Kalman Filter is used. The dynamics of parameter changing is also assumed as constant

(37) $$w_{k+1}=w_{k}+\omega_{k}^{w},$$

where $\omega_{k}^{w}$ is a noise with variance given by matrix $Q_{k}^{w}$. The equations of the filter KF*w* are very similar to filter KF*o*, and the difference is only in the model replica part:

(38) $$\begin{array}{ccc} {\tilde{w}}_{k} & = & {\hat{w}}_{k-1}, \end{array}$$

(39) $$\begin{array}{ccc} {\tilde{P}}_{k}^{w} & = & {\hat{P}}_{k-1}^{w}+Q_{k-1}^{w}, \end{array}$$

(40) $$\begin{array}{ccc} {\tilde{W}}_{k} & = & \left[\begin{array}{cc} {\tilde{w}}_{k} & {\tilde{w}}_{k}\pm {\left(\sqrt{\left(L+\lambda \right){\tilde{P}}_{k}^{w}}\right)}_{i} \end{array}\right], \end{array}$$

(41) $$\begin{array}{ccc} \Delta^{{\hat{\alpha }}_{k-1}}{\tilde{\chi }}_{k,i}^{w} & = & A\left({\tilde{W}}_{k,i}\right){\hat{x}}_{k-1}+Bu_{k-1}, \end{array}$$

(42) χ˜k,iw=hα^k−1Δα^k−1χ˜k,iw−∑j=1k(−1)jα^k−1jx^k−j.

Resume, the TEA consists of three sub-filters whose required separate sets of parameters and initial conditions. Parameters of the order estimation filter KF*o* are denoted with upper index ^*o*^ (e.g., ${\tilde{P}}_{k}^{o}$, $Q_{k-1}^{o}$), parameters of KF*w* are denoted with upper index ${}^{w}$ (e.g., ${\tilde{P}}_{k}^{w}$, $Q_{k-1}^{w}$) and parameters of KF*x* are without upper index. A detailed description of the Triple Estimation Algorithm is presented in [40].

## 4. Identification and Analysis of Fractional Order Noise Parameters

Before we apply the Triple Estimation algorithm to real plant data (noises estimation of MEMS sensor), we will present the results of some numerical experiments for constant and FVO systems. The one state variable discrete state-space system, used in numerical experiments, is given as follows:

(43) Δk+1αk+10Axk+1=fxk+uk+ωk,

(44) xk+1=hαk+1Δk+1αk+10xk+1−∑j=1k+1(−1)jαk+1jxk−j+1,

(45) $$\begin{array}{ccc} y_{k} & = & x_{k}+\nu_{k}\;. \end{array}$$

### 4.1. Analysis of Fractional Constant and Variable Order System with Input Signal Known

This section contains a validation of the TEA for analysis and identification of fractional constant and variable order systems. Sets of numerical examples show the capability of the state, parameter and order estimation for known input signal to be a Gaussian noise. The problem formulated in Examples 1–3 is as follows: Estimate the state, order, and parameter of the fractional order system described by (43)–(45) with known input signal being the Gaussian noise. All numerical examples were conducted in Matlab/Simulink environment based on Fractional Variable Order Toolbox [43] with sampling time equals to h=0.001 s. It is worth noticing that the final results of state, order, and parameter estimation depend on Triple Estimation filters’ parameters (KF*x*, KF*o*, and KF*w*) and should be individually adjusted.

The parameters of TEA applied in Example 1 are as following:

- Noises parameters
  $$E\left[\omega \omega^{T}\right]=10^{-4},$$
  $$E\left[\nu \nu^{T}\right]=10^{-3},$$
- Parameters of KF*x* filter
  $$P_{0}=\left[\begin{array}{c} 0.01 \end{array}\right],\;Q_{0}=\left[\begin{array}{c} 10^{-4} \end{array}\right],$$
  $$x_{0}=\left[\begin{array}{c} 0 \end{array}\right],\;R=\left[\begin{array}{c} 10^{-3} \end{array}\right],$$
- Parameters of KF*o* filter
  $$P_{0}^{o}=\left[\begin{array}{c} 0.01 \end{array}\right],\;Q_{0}^{o}=\left[\begin{array}{c} 0.1 \end{array}\right],$$
  $$\alpha_{0}=\left[\begin{array}{c} 1 \end{array}\right],\;R^{o}=\left[\begin{array}{c} 10^{-3} \end{array}\right],\;A=1,\;B=2,\;\delta^{o}=0.5,$$
- Parameters of KF*w* filter
  $$P_{0}^{w}=\left[\begin{array}{c} 0.01 \end{array}\right],\;Q_{0}^{w}=\left[\begin{array}{c} 0.1 \end{array}\right],$$
  $$w_{0}=\left[\begin{array}{c} 0 \end{array}\right],\;R^{w}=\left[\begin{array}{c} 10^{-3} \end{array}\right],\;A=1,\;B=2,\;\delta^{w}=0.5.$$

An identification of fractional constant order system based on TEA is presented in Example 1.

**Example** **1.** *Let us consider the DFVOSS A-type system given by* (43)–(45)*, where*

(46) $$A=f=-1,\;B=1,\;C=1,\;\alpha_{k}=0.35,\;u_{k}∼N\left(0,1\right).$$

**Figure 2:**
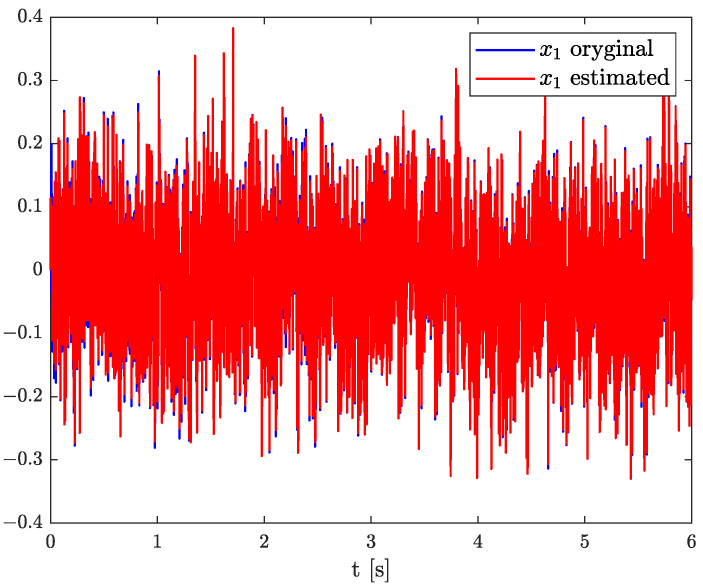
Original and estimated state variable from Example 1.

**Figure 3:**
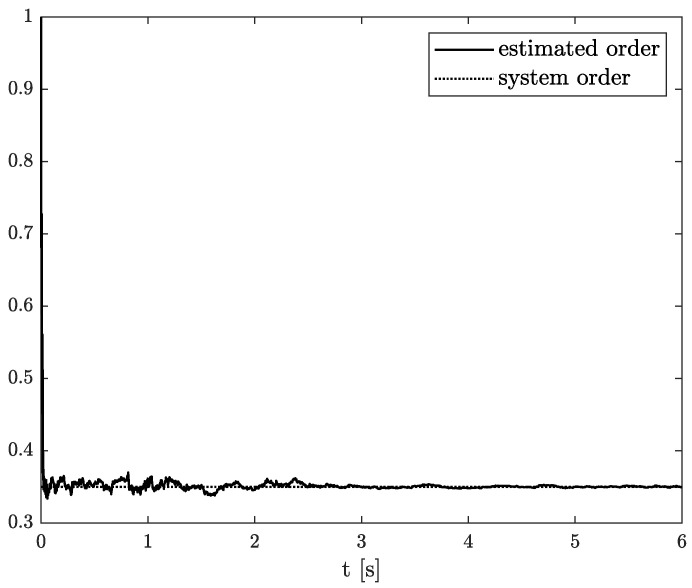
Original and estimated order from Example 1.

**Figure 4:**
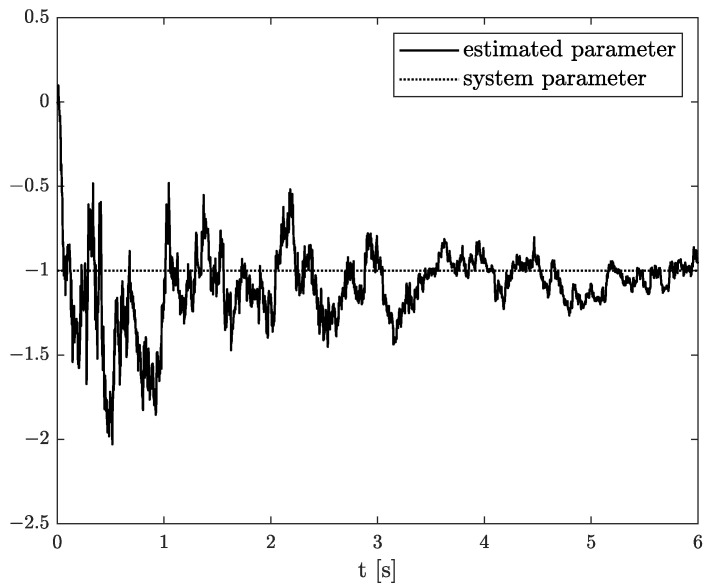
Original and estimated parameter from Example 1.

Numerical results of state, order, and parameter estimation are presented in Figure 2, Figure 3 and Figure 4, respectively. As it can be seen in Figure 2, the state estimation overlaps the original one with high accuracy. Similarly, we can distinguish a high accuracy of order and parameter estimation presented in Figure 3 and Figure 4. The order estimation reaches the original one practically in unnoticeable time. The discrepancy between system parameter and its estimation decays. Due to the initial order value, the estimated order relatively slowly points out the simulated one. The desired simulation value is reached out with decreasing fluctuations at 5 s. In conclusion, the state, order, and parameter estimation using the TEA precisely reflect simulation values. The filter’s parameters can adjust the quality of achieved results individually. It mainly depends on ongoing cases cause the TEA can be treated as a convenient tool for identifying and analysing FO systems and noises. Then the rate of precisely achieved results does not play a significant role. Once identified, FO models can be applied to filter out useless data.

Temperature sensitivity is one of the most significant effects, exerting a huge impact on inertial measurement unit’s noises built-in MEMS technology. Temperature-varying can force the modification in order values during the process, and it is worth to validate the TEA for this case. Therefore, the next examples are devoted to state, order, and parameter estimation of FVO systems. The main difference between these two examples occurs in the order function. In Example 2, the original order function is described by linear function, and in Example 3 reference order function has a parabolic character.

The parameters of TEA applied in Examples 2 and 3 are as follows:

- Noises parameters
  $$E\left[\omega \omega^{T}\right]=10^{-4},$$
  $$E\left[\nu \nu^{T}\right]=10^{-5},$$
- Parameters of KF*x* filter
  $$P_{0}=\left[\begin{array}{c} 0.01 \end{array}\right],\;Q_{0}=\left[\begin{array}{c} 10^{-4} \end{array}\right],$$
  $$x_{0}=\left[\begin{array}{c} 0 \end{array}\right],\;R=\left[\begin{array}{c} 10^{-5} \end{array}\right],$$
- Parameters of KF*o* filter
  $$P_{0}^{o}=\left[\begin{array}{c} 0.1 \end{array}\right],\;Q_{0}^{o}=\left[\begin{array}{c} 0.001 \end{array}\right],$$
  $$\alpha_{0}=\left[\begin{array}{c} 0.2 \end{array}\right],\;R^{o}=\left[\begin{array}{c} 10^{-5} \end{array}\right],\;A=1,\;B=2,\;\delta^{o}=0.5,$$
- Parameters of KF*w* filter
  $$P_{0}^{w}=\left[\begin{array}{c} 0.01 \end{array}\right],\;Q_{0}^{w}=\left[\begin{array}{c} 0.001 \end{array}\right],$$
  $$w_{0}=\left[\begin{array}{c} -0.5 \end{array}\right],\;R^{w}=\left[\begin{array}{c} 10^{-5} \end{array}\right],\;A=1,\;B=2,\;\delta^{w}=0.5.$$

**Example** **2.** *Let us consider the DFVOSS A-type system given by* (43)–(45)*, where*

(47) $$A=f=-1,\;B=1,\;C=1,u_{k}∼N\left(0,0.1\right),\begin{array}{cc} \alpha_{k}=0.2+0.1kh & for\;k=1,2,3,\ldots . \end{array}.$$

**Figure 5:**
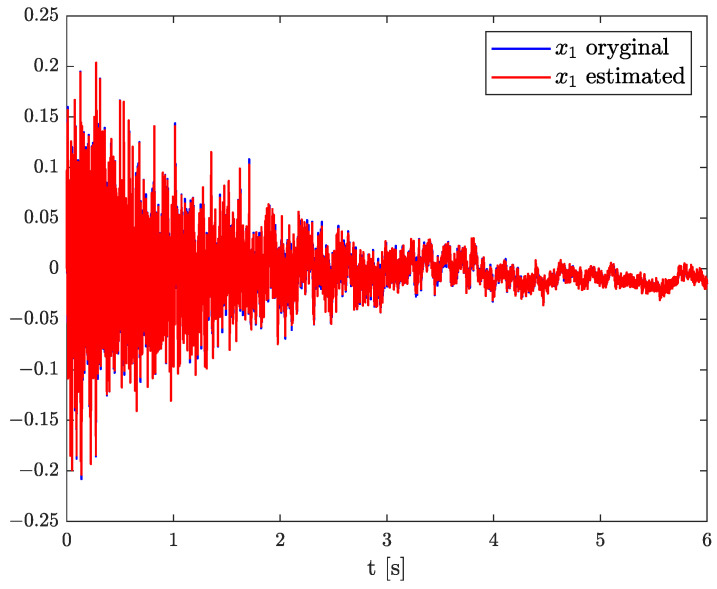
Original and estimated state variable from Example 2.

**Figure 6:**
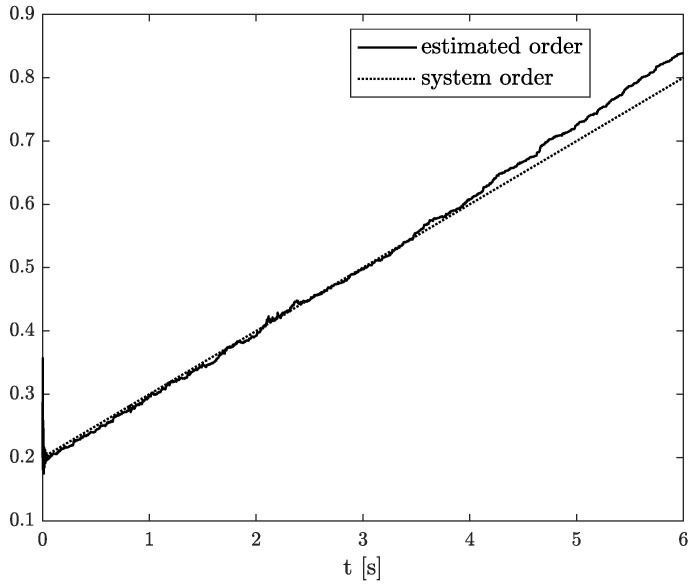
Original and estimated order from Example 2.

**Figure 7:**
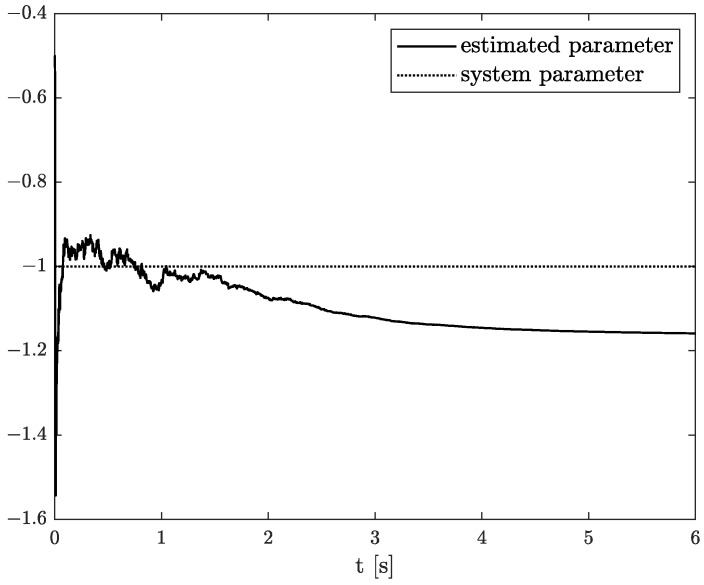
Original and estimated parameter from Example 2.

The example results of state, order, and parameter estimation are shown in Figure 5, Figure 6 and Figure 7, respectively. In this case, the estimated order precisely overlaps the original one until 4 s, and after that, a minor discrepancy appears. Additionally, there is a small underestimation between the original and algorithm’s parameter. However, both estimated values (order and parameter) lead to high accuracy of state estimation (see Figure 5). The example shows the behaviour of TEA in a wide range of order values while a linear function represents the order function. The estimated order coincides with the simulation one very well. It is a significant advantage of the TEA because the temperature effects influence noise components such as random walk errors or bias instability and directly into the model’s order. It shows that despite unexpected disturbances appearing in the FVO systems, the TEA can be applied for their identification and analysis.

**Example** **3.** *Let us consider the DFVOSS A-type system given by* (43)–(45)*, where*

(48) $$A=f=-1,\;B=1,\;C=1,\;u_{k}∼N\left(0,0.1\right),\begin{array}{cc} \alpha_{k}=0.2+0.05\cdot {\left(kh\right)}^{2} & for\;k=1,2,3,\ldots . \end{array}.$$

**Figure 8:**
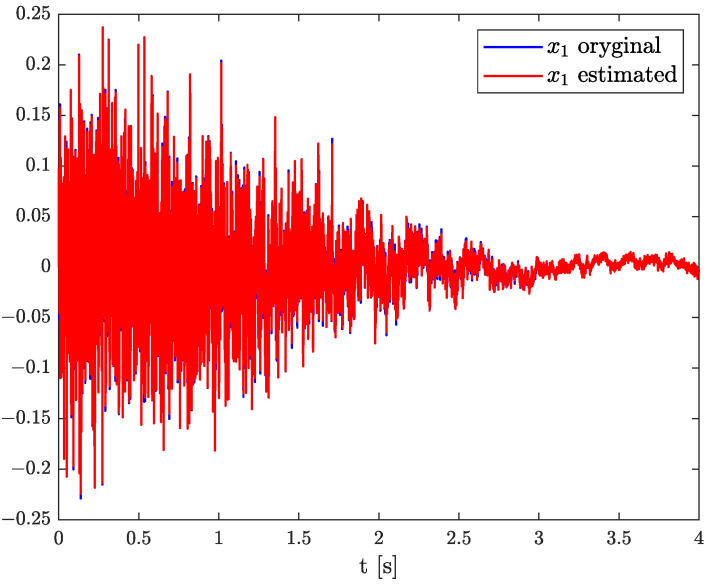
Original and estimated state variable from Example 3.

**Figure 9:**
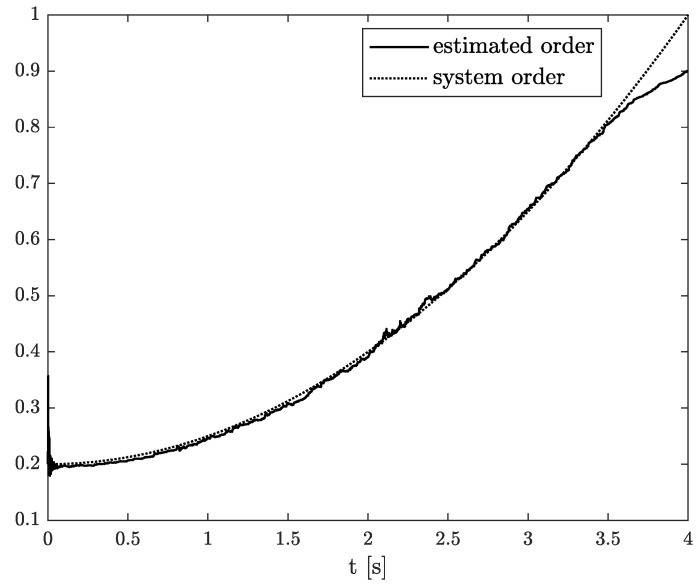
Original and estimated order from Example 3.

**Figure 10:**
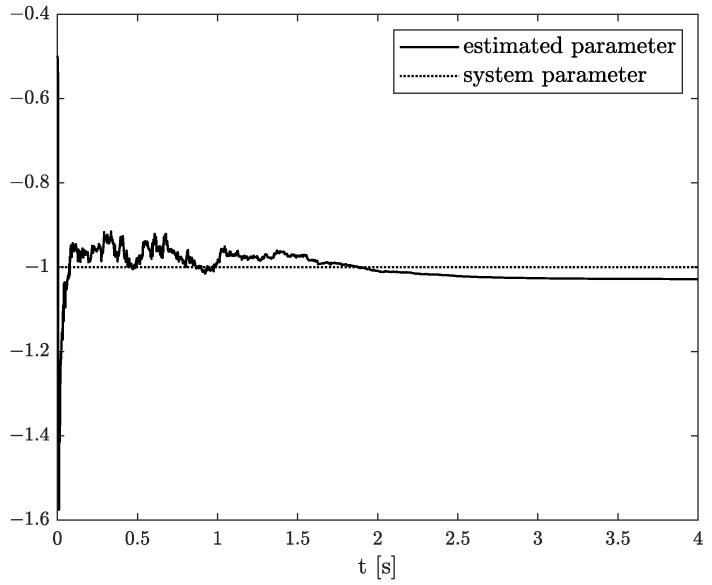
Original and estimated parameter from Example 3.

This time, the results of state, order, and parameter estimation are presented in Figure 8, Figure 9 and Figure 10, respectively. Moreover, the order-varying is forced by parabolic function, and despite of it, high accuracy of state, order, and parameter estimation is achieved. The accelerometers are measurement devices that work well while not being subjected to external forces or disturbances. They are susceptible, and additional actions can imply undesirable errors. It is an essential issue while double integral is needed to specify the position in inertial navigation systems (INS) and any error accumulated inaccuracies very fast. Hence, in this example, we show the situation when the order function changes its value more quickly than in Example 2. It conveys the possibilities of TEA against additional errors.

The results directly coming from Examples 1–3 show that TEA can be successfully used to estimate state, order, and parameter for fractional constant and variable order systems with known input signal. Especially, the capability of FVO systems analysis seems to be very important during noise modelling of MEMS. It is caused by the fact that the temperature effect can change the order of noise. Hence, the time-varying order occurring in the noise model makes it much more flexible and reflects its random walk errors.

The average execution time of TEA equals 495 s for a set of 6000 combined samples of state, order, and parameter estimation, which corresponds to Examples 1 and 2. The time-consuming tests were conducted on PC with Intel Core i7-5500U CPU, 2.4 GHz, RAM 8 GB and Matlab version 2021b 64 bit.

### 4.2. Identification without Input Signal Knowledge

When an input signal is not measured, the identification process can differ from desired values or order and system parameter. This can be explained by the fact that in practice, the system noise can have some unknown dynamical correlation of some order and parameter. Let us assume the fractional noise system equation in the following form:

(49) $$\Delta_{1}^{\alpha }x_{k+1}=f_{1}x_{k}+\omega_{k}^{\prime },$$

where $\omega_{k}^{\prime }$ is a system noise also containing the fractional order dynamical correlation described by the following relation:

(50) $$\Delta_{2}^{\alpha }\omega_{k+1}^{\prime }=f_{2}\omega_{k}^{\prime }+\omega_{k},$$

where $\omega_{k}$ is assumed to be noise without dynamical correlation.

By combining both equations we obtain

(51) $$\Delta_{1}^{\alpha }x_{k+1}-\frac{1}{f_{2}}\Delta_{2}^{\alpha }\omega_{k+1}^{\prime }=f_{1}x_{k}-\frac{1}{f_{2}}\omega_{k}.$$

As we can see, this dynamical correlation can have a direct effect on estimated order and system parameter in the estimation process, which can make obtained estimation results different from those assumed in numerical models, because they take into consideration also the dynamical correlation of the source noise. However, it will not be a problem in estimation of real plant noise because the aim of estimation is to find the most appropriate model with the assumption that the source noise is without dynamical correlation.

## 5. Identification and Analysis of MEMS Accelerometer’s Noises

This section contains the experimental results of noises modelling for the 3-axes accelerometer being the part of SparkFun MPU9250 Inertial Measurement Unit (IMU) built-in MEMS technology. This is a very popular MEMS, and there exist plenty of robotics projects where it is a base for many Attitude and Heading Reference System (AHRS) and INS implementations. Generally, accelerometers are very sensitive, and they are characterised by high frequencies noises. All of these can have a significant impact on final project results. This is why we decided to apply the TEA for the noises modelling of the accelerometer as part of MPU9250 breakout board.

Overall, the MPU9250 unit is a 9 degree of freedom MEMS with 3 accelerometer’s axes, 3 gyroscope’s axes, and 3 magnetometer’s axes. The MPU9250 breakout board runs on 3.3 VDC and contains I^2^C and SPI communication protocols.

### 5.1. Experimental Setup

**Figure 11:**
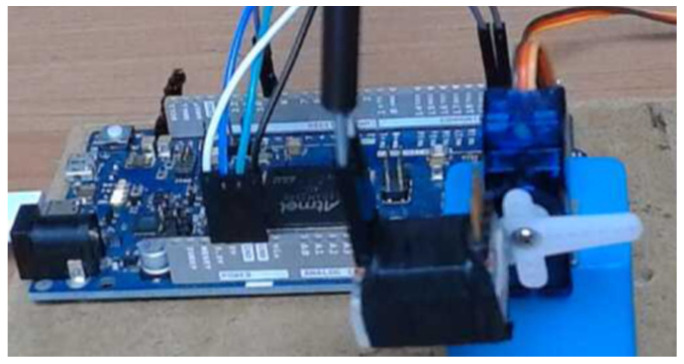
The real view of experimental setup with Arduino Due development board and MPU9252 IMU mounted on a shaft of servo motor in lock position.

The experimental approach was divided into two main parts. The first one was devoted to data collection, and the second one was related to their identification based on TEA. Therefore, the first phase can be represented by experimental setup shown in Figure 11. Its main parts are the Arduino Due development board and MPU9250 breakout board. The I^2^C communication protocol was used for data transmission between them. Additionally, the accelerometer’s range was set to 8 g, and measurement’s data were collected in the Arduino IDE environment for stationary located IMU, with sampling time h=0.01 s. The lock accelerometer position allows gathering its noises corrupted by constant gravity components. In the second phase of the experiment, the calculated mean value of data for each axis was subtracted from its measurement to have the pure noise corresponding to a particular accelerometer axis. Then, post-processing data for each axis without mean value were adapted as *x*-axis, *y*-axis, and *z*-axis noises in the Matlab environment. Having such prepared noises data and due to the fact that their mathematical models are independent from each other, we decided to apply the TEA separately for each axis. In fact, the state, parameter, and order estimation results were achieved for each axis separately under the same TEA configuration parameters except covariances *R*, $R^{o}$, and $R^{w}$ adjusted according to noise measurement corresponding to each axis.

To summarize, in the experimental setup, the problem is formulated as follows: Estimate noise, its order and parameter for *x*, *y* and *z*-axes separately, with no input signal knowledge. Therefore, the real noises data originating from the accelerometer do not take part in the TEA, but they are shown only for validation purposes.

The parameters of TEAs for each acceleromenter’s axis are as the following:

- Parameters of KF*x* filter
  $$P_{0}=\left[\begin{array}{c} 0.01 \end{array}\right],\;Q_{0}=\left[\begin{array}{c} 0.01 \end{array}\right],$$
  $$x_{0}=\left[\begin{array}{c} 0 \end{array}\right],\;R=\left\{\begin{array}{cc} 4\cdot 10^{-4} & \;\mathrm{for}\;x-\mathrm{axis}\mathrm{noise}\\ 3.9\cdot 10^{-4} & \;\mathrm{for}\;y-\mathrm{axis}\mathrm{noise}\\ 11\cdot 10^{-4} & \;\mathrm{for}\;z-\mathrm{axis}\mathrm{noise} \end{array}\right.$$
- Parameters of KF*o* filter
  $$P_{0}^{o}=\left[\begin{array}{c} 0.01 \end{array}\right],\;Q_{0}^{o}=\left[\begin{array}{c} 0.1 \end{array}\right],$$
  $$\alpha_{0}=\left[\begin{array}{c} 1 \end{array}\right],\;A=1,\;B=2,\;\delta^{o}=0.5,\;R^{o}=\left\{\begin{array}{cc} 4\cdot 10^{-4} & \;\mathrm{for}\;x-\mathrm{axis}\mathrm{noise}\\ 3.9\cdot 10^{-4} & \;\mathrm{for}\;y-\mathrm{axis}\mathrm{noise}\\ 11\cdot 10^{-4} & \;\mathrm{for}\;z-\mathrm{axis}\mathrm{noise} \end{array}\right.$$
- Parameters of KF*w* filter
  $$P_{0}^{w}=\left[\begin{array}{c} 0.01 \end{array}\right],\;Q_{0}^{w}=\left[\begin{array}{c} 0.1 \end{array}\right],$$
  $$w_{0}=\left[\begin{array}{c} 0 \end{array}\right],\;A=1,\;B=2,\;\delta^{w}=0.5,\;R^{w}=\left\{\begin{array}{cc} 4\cdot 10^{-4} & \;\mathrm{for}\;x-\mathrm{axis}\mathrm{noise}\\ 3.9\cdot 10^{-4} & \;\mathrm{for}\;y-\mathrm{axis}\mathrm{noise}\\ 11\cdot 10^{-4} & \;\mathrm{for}\;z-\mathrm{axis}\mathrm{noise}. \end{array}\right.$$

### 5.2. Experimental Results

**Figure 12:**
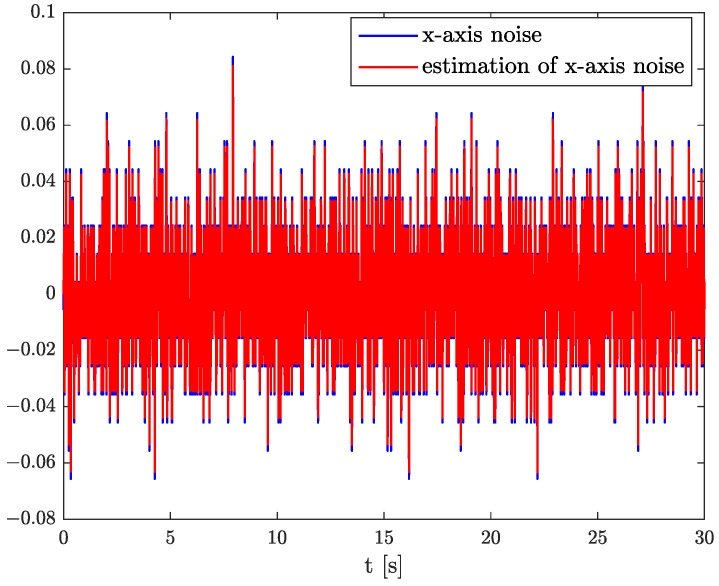
Original and estimation of *x*-axis noise.

**Figure 13:**
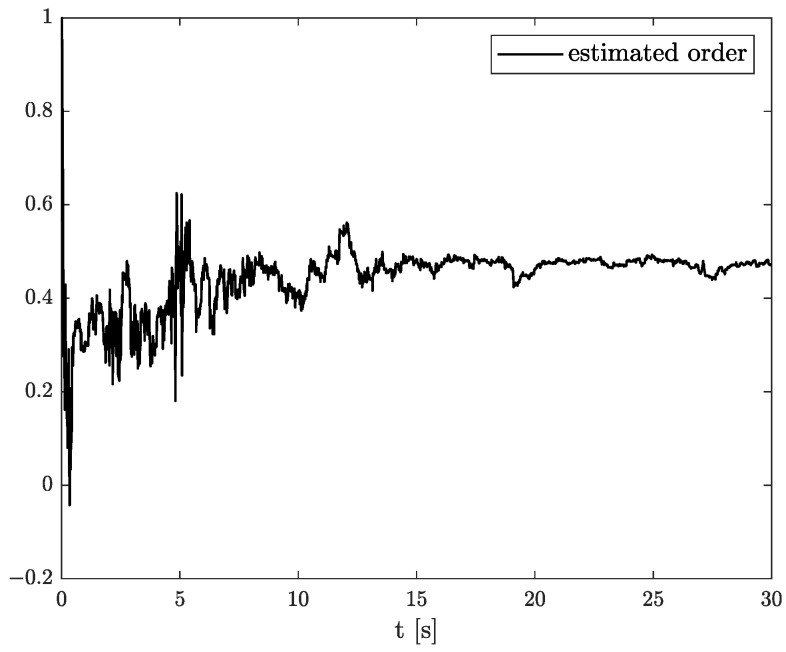
Order estimation for *x*-axis noise.

**Figure 14:**
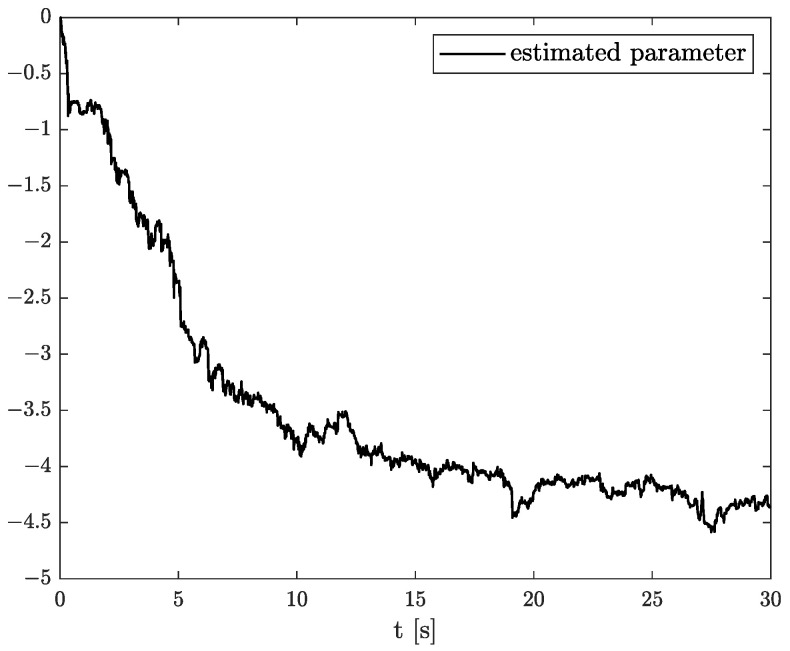
Parameter estimation for *x*-axis noise.

**Figure 15:**
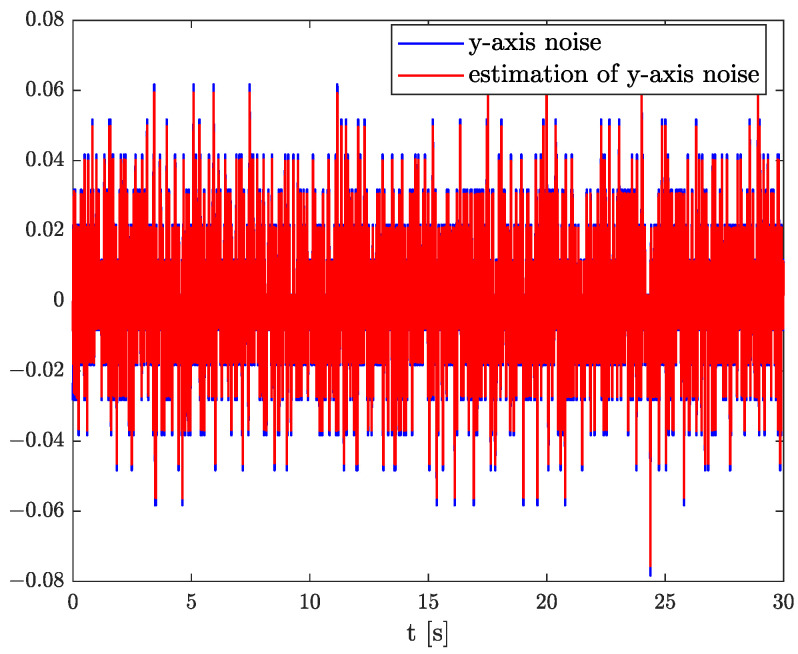
Original and estimation of *y*-axis noise.

**Figure 16:**
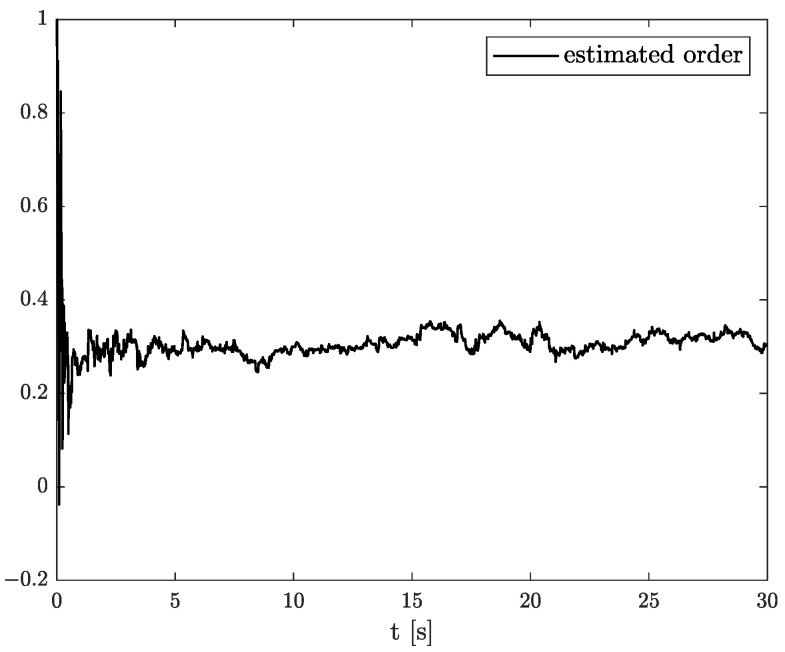
Order estimation for *y*-axis noise.

**Figure 17:**
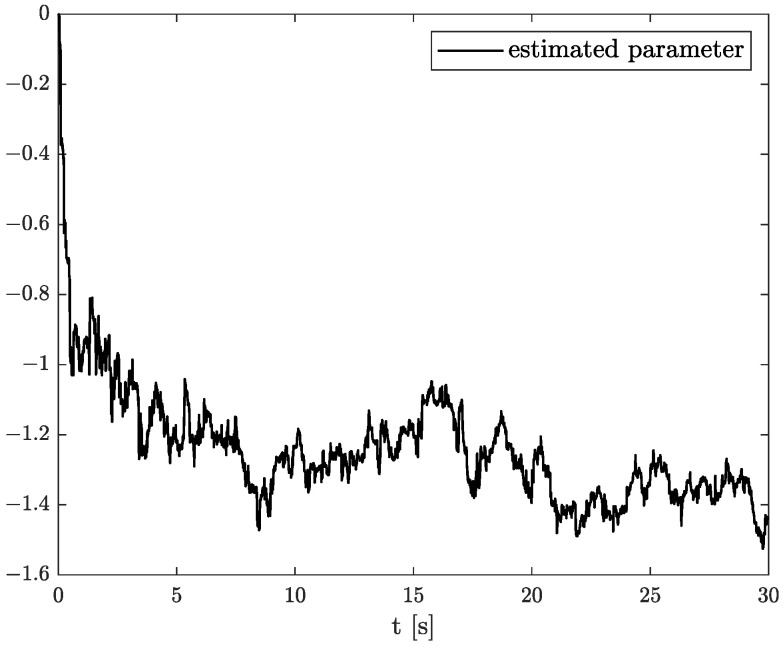
Parameter estimation for *y*-axis noise.

**Figure 18:**
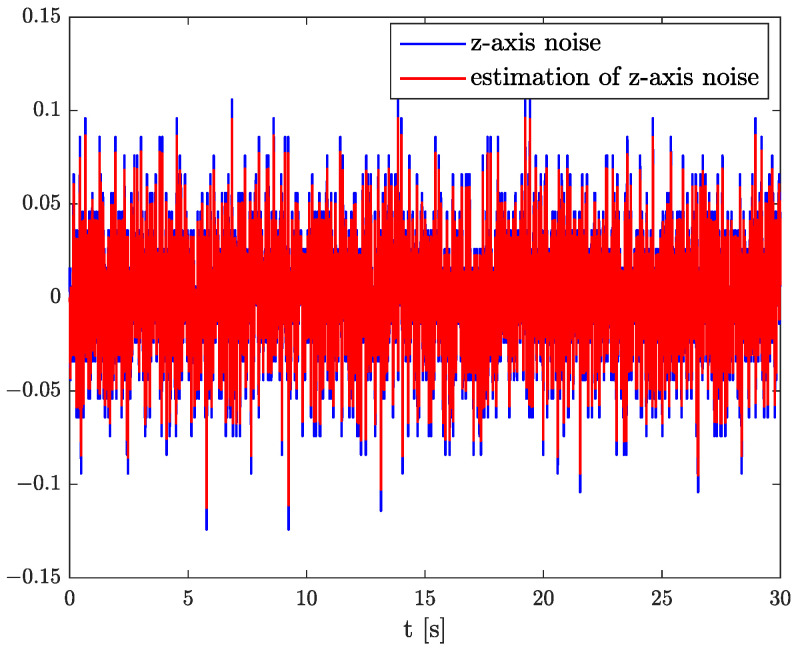
Original and estimation of *z*-axis noise.

**Figure 19:**
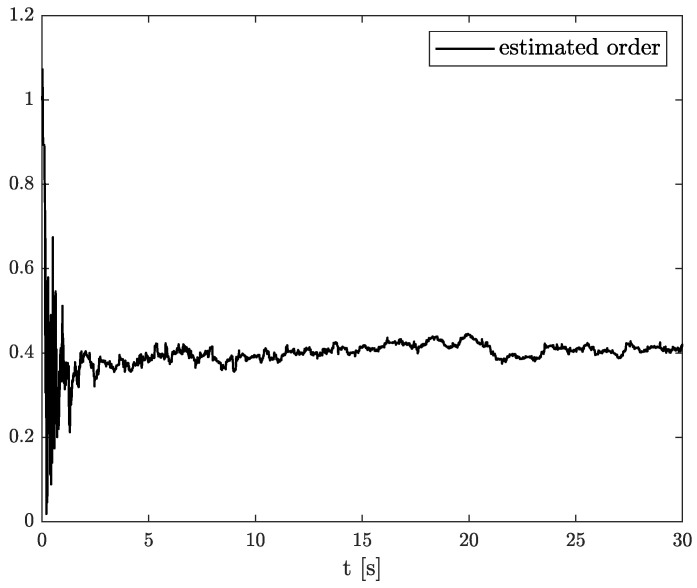
Order estimation for *z*-axis noise.

**Figure 20:**
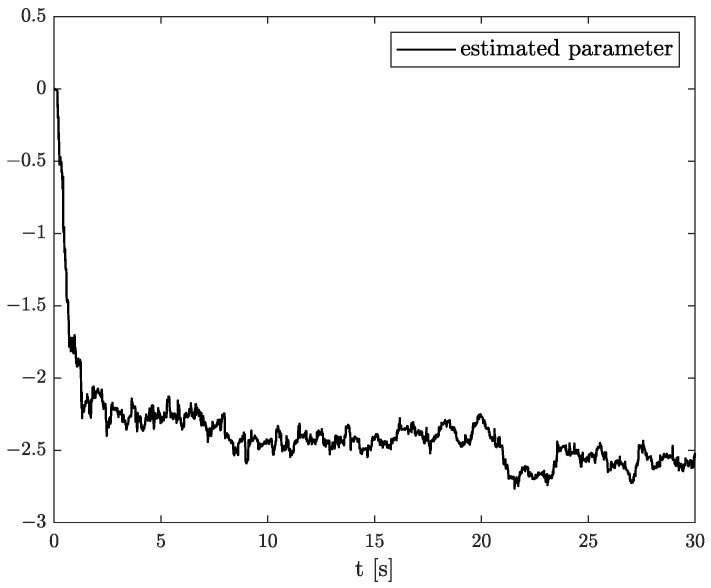
Parameter estimation for *z*-axis noise.

In the results of the experimental research, we have obtained the three sets of plots corresponding to *x*, *y*, and *z*-axes of accelerometer’s noises. Therefore, the first set of plots for noise, order, and parameter estimation related to *x*-axis are presented in Figure 12, Figure 13 and Figure 14, respectively. The estimated order rapidly goes from the initial value to around 0.3 and then, with minor fluctuations, raises to the neighbourhood of 0.4 at time 15 s, while parameter estimation slowly decreases to −4 and reaches this value after 15 s on the plot. We can observe that after 15 s, the order and parameter are relatively stabilised. The plots concerning to *y*-axis noise together with order and parameter estimation can be found in Figure 15, Figure 16 and Figure 17. This time, the estimated order achieve its stabilised value around 0.3, and the curve reaches it approximately in 2 s. Then, the fluctuations are relatively slight during the whole estimation process. The estimated parameter goes to −1 rapidly, in approximately 2 s and then with small oscillations tends to a neighbourhood of −1.4. At the end, the experimental results of noise modelling for *z*-axis are presented in Figure 18. Additionally, its order and parameter estimation are shown in Figure 19 and Figure 20, respectively. For *z*-axis noise, the estimated order reaches its stabilised value around 0.4 at time 3 s and then tries to keep it. The order curve is relatively slight with minor fluctuations, while the estimated parameter starting with the initial value tends to −2.5, and the plot follows it beginning with 4 s.

Comparing the original noises for *x*, *y*, and *z*-axes to their estimation results presented in Figure 12, Figure 15, and Figure 18 confirm the fractional dynamics of MPU9250 accelerometer’s noises and the high quality of applied method. Moreover, we can see that order values for each axis noise are similar, and they tend to fractional constant values. Hence, we can conclude that the temperature effects did not impact the accelerometer’s random walk errors too much.

The average execution time of TEA equals 122 s for a set of 3000 combined samples of state, order, and parameter estimation. This time corresponds to each accelerometer’s axis, separately.

## 6. Conclusions

The paper presents the results of the Triple Estimation Algorithm application to estimate fractional order noises. The algorithm can simultaneously estimate state variables, system parameters, and FVO of the noises. Presented numerical experiments show the ability of the algorithm to determine the variable order for the FVO noise. Simultaneous estimation can be an essential feature to test if unknown noise obtained experimentally is stationary or time-varying. As it is known, the low-cost MEMS used in many projects done by hobbyists are temperature sensitive. In this case, the capability of FVO noise analysis can handle its unexpected influence on final results and raise their accuracy. Comparing the real accelerometer noises to their estimates, it could be seen that the TEA was successfully applied to noises modelling of 3-axes accelerometer being the part of MPU9250 breakout board. The experimentally obtained data confirm the fractional character of investigated sensor’s noises, and the estimated order values for *x*, *y* and *z*-axes are similar to each other. Carrying out tests in examples and experiments acknowledges that the TEA can be treated as an effective method for simultaneously estimating states, variable order, and system parameters. Moreover, the main advantage of the proposed algorithm against other existing techniques is that it obtains the unknown system parameters and variable order at once, giving the ability to analyse the time-correlated noise. The obtained results can be extended to examine external factors (like temperature) on the noise order and system parameters. Another possible area of use for the obtained results is the development of estimation algorithms for AHRS or inertial navigation, which will use estimated values of fractional noises. Numerical simulations show that accurate results of state, order, and parameter estimation given by TEA come with the complexity of the algorithm which is relatively high and time consuming.

## Data Availability

Not applicable.

## References

[1] Vasudev A., Bhansali S., Inmann A., Hodgins D. (2013). Microelectromechanical systems (MEMS) for in vivo applications. Implantable Sensors Systems for Medical Applications.

[2] Panescu D. (2006). MEMS in medicine and biology. IEEE Eng. Med. Biol. Mag..

[3] Deisingh A. (2003). MEMS technology in analytical chemistry. Analyst.

[4] Leclerc J. (2007). MEMs for aerospace navigation. IEEE Aerosp. Electron. Syst. Mag..

[5] Classen J., Frey J., Kuhlmann B., Ernst P., Valldorf J., Gessner W. (2007). MEMS gyroscopes for automotive applications. Advanced Microsystems for Automotive Applications 2007.

[6] Goel A., Ul Islam A., Ansari A., Kouba O., Bernstein D.S. (2021). An Introduction to Inertial Navigation From the Perspective of State Estimation [Focus on Education]. IEEE Control. Syst. Mag..

[7] Yazdi N., Ayazi F., Najafi K. (1998). Micromachined inertial sensors. Proc. IEEE.

[8] Chen F., Fei J., Xue Y. (2021). Double Recurrent Perturbation Fuzzy Neural Network Fractional-Order Sliding Mode Control of Micro Gyroscope. IEEE Access.

[9] Mohd-Yasin F., Nagel D.J., Korman C.E. (2010). Noise in MEMS. Meas. Sci. Technol..

[10] Krysko V., Awrejcewicz J., Yakovleva T., Kirichenko A., Szymanowska O., Krysko V. (2019). Mathematical modeling of MEMS elements subjected to external forces, temperature and noise, taking account of coupling of temperature and deformation fields as well as a nonhomogenous material structure. Commun. Nonlinear Sci. Numer. Simul..

[11] Qiao Y., Arabi M., Xu W., Zhang H., Abdel-Rahman E.M. (2021). The impact of thermal-noise on bifurcation MEMS sensors. Mech. Syst. Signal Process..

[12] Romanovas M., Klingbeil L., Traechtler M., Manoli Y. (2009). Application of fractional sensor fusion algorithms for inertial MEMS sensing. Math. Model. Anal..

[13] Samko S., Kilbas A., Maritchev O. (1987). Fractional Integrals and Derivative. Theory and Applications.

[14] Miller K., Ross B. (1993). An Introduction to the Fractional Calculus and Fractional Differenctial Equations.

[15] Monje C.A., Chen Y., Vinagre B.M., Xue D., Feliu V. (2010). Fractional-Order Systems and Controls.

[16] Podlubny I. (1999). Fractional Differential Equations.

[17] Magin R., Ortigueira M.D., Podlubny I., Trujillo J. (2011). On the fractional signals and systems. Signal Process..

[18] Kilbas A.A., Srivastava H.M., Trujillo J.J. (2006). Theory and Applications of Fractional Differential Equations, Volume 204 (North-Holland Mathematics Studies).

[19] Das S. (2011). Introduction to Fractional Calculus. Functional Fractional Calculus.

[20] Anastassiou G.A. (2021). Generalized Fractional Calculus.

[21] Yang X.J. (2019). General Fractional Derivatives: Theory, Methods and Applications.

[22] Tarasov V.E. (2018). Generalized Memory: Fractional Calculus Approach. Fractal Fract..

[23] Sierociuk D., Dzielinski A., Sarwas G., Petras I., Podlubny I., Skovranek T. (2013). Modelling heat transfer in heterogeneous media using fractional calculus. Philos. Trans. R. Soc. A Math. Phys. Eng. Sci..

[24] Sakrajda P., Sierociuk D., Babiarz A., Czornik A., Klamka J., Niezabitowski M. (2017). Modeling Heat Transfer Process in Grid-Holes Structure Changed in Time Using Fractional Variable Order Calculus. Theory and Applications of Non-Integer Order Systems.

[25] Reyes-Melo M., Martinez-Vega J., Guerrero-Salazar C., Ortiz-Mendez U. Application of fractional calculus to modelling of relaxation phenomena of organic dielectric materials. Proceedings of the 2004 IEEE International Conference on Solid Dielectrics.

[26] Dzielinski A., Sierociuk D., Sarwas G. (2010). Some applications of fractional order calculus. Bull. Pol. Acad. Sci. Tech. Sci..

[27] Ortigueira M.D., Valério D. (2020). Fractional Signals and Systems.

[28] Sheng H., Chen Y., Qiu T. (2012). Fractional Processes and Fractional-Order Signal Processing.

[29] Muresan C.I., Birs I.R., Dulf E.H., Copot D., Miclea L. (2021). A Review of Recent Advances in Fractional-Order Sensing and Filtering Techniques. Sensors.

[30] Sierociuk D., Malesza W., Macias M. (2015). Derivation, interpretation, and analog modelling of fractional variable order derivative definition. Appl. Math. Model..

[31] Sierociuk D., Malesza W., Macias M. (2015). On the Recursive Fractional Variable-Order Derivative: Equivalent Switching Strategy, Duality, and Analog Modeling. Circuits Syst. Signal Process..

[32] Macias M., Sierociuk D. An alternative recursive fractional variable-order derivative definition and its analog validation. Proceedings of the ICFDA’14 International Conference on Fractional Differentiation and Its Applications.

[33] Sierociuk D., Ziubinski P. (2015). Variable order fractional Kalman filters for estimation over lossy network. Lect. Notes Electr. Eng..

[34] Ziubinski P., Sierociuk D. Improved fractional Kalman filter for variable order systems. Proceedings of the ICFDA’14 International Conference on Fractional Differentiation and Its Applications.

[35] Wyss W. (1991). Fractional noise. Found. Phys. Lett..

[36] Chen Y., Sun R., Zhou A., Zaveri N. (2008). Fractional order signal processing of electrochemical noises. J. Vib. Control..

[37] Sierociuk D., Ziubinski P. (2014). Fractional order estimation schemes for fractional and integer order systems with constant and variable fractional order colored noise. Circuits Syst. Signal Process..

[38] Bai Y., Wang X., Jin X., Su T., Kong J., Zhang B. (2020). Adaptive filtering for MEMS gyroscope with dynamic noise model. ISA Trans..

[39] Ziubinski P., Sierociuk D. Fractional order noise identification with application to temperature sensor data. Proceedings of the 2015 IEEE International Symposium on Circuits and Systems (ISCAS).

[40] Sierociuk D., Macias M. (2021). Triple Estimation of Fractional Variable Order, Parameters, and State Variables Based on the Unscented Fractional Order Kalman Filter. Sensors.

[41] Sierociuk D., Malesza W. (2017). Fractional variable order discrete-time systems, their solutions and properties. Int. J. Syst. Sci..

[42] Haykin S. (2001). Kalman Filtering and Neural Networks.

[43] Sierociuk D. (2012). Fractional Variable Order Derivative Simulink Toolkit. MATLAB Central File Exchange. http://www.mathworks.com/matlabcentral/fileexchange/38801-fractional-variable-order-derivative-simulink-toolkit.

